# Decoding Readiness for Clinical Practicum: Undergraduate Nursing Students’ Perspectives, Clinical Evaluations, and Comparative Curriculum Variations

**DOI:** 10.3390/nursrep15060204

**Published:** 2025-06-05

**Authors:** Imad Maalouf, Wafaa El Zaatari

**Affiliations:** 1 Nursing Department, Fatima College of Health Sciences, Al Ain 999041, United Arab Emirates; imad.maalouf@fchs.ac.ae; 2 College of Interdisciplinary Studies, Zayed University, Dubai 19282, United Arab Emirates

**Keywords:** nursing students, readiness for clinical practicum, nursing education

## Abstract

**Background:** Nursing students’ readiness for clinical practicums is critical to nursing education. Concerns about students’ preparedness for clinical courses have emerged due to increased student-to-educator ratios and limited hands-on practice time. Moreover, feedback from clinical instructors reveals that many student nurses lack the necessary knowledge and skills for patient care, thereby raising questions about their readiness for clinical practicum. **Purpose:** This study investigates undergraduate nursing students’ readiness for clinical practicum in the UAE by examining their perspectives, the variation in clinical study plans across different contexts, and the evidence gathered from clinical evaluations. **Methodology:** A case study design was adopted, utilizing semi-structured interviews with 13 nursing students from a UAE nursing college. Additionally, two types of document analysis were conducted. First, 11 nursing curricula from high-ranking universities were analyzed to compare whether students received adequate laboratory courses for their clinical practicum. Second, 217 clinical evaluation reports from third- and fourth-year nursing students across 4 campuses of the UAE nursing college were reviewed. **Findings:** The study identified two key themes from the interviews: incomplete readiness for clinical practicum and the factors contributing to this incomplete readiness. Document analysis revealed that, unlike many American and Australian institutions, most universities lacked co-requisite laboratory courses. Clinical evaluation reports highlighted that some students, particularly in their fourth year, were inadequately prepared for clinical practice due to deficiencies in both clinical skills and theoretical knowledge. **Conclusions:** The findings indicate that many nursing students felt only partially prepared for their practicum, negatively impacting their confidence and competency. Moreover, adopting the American and Australian approach of pairing practicum courses with laboratory courses may better prepare students for clinical practicum. Recommendations for future research have been outlined.

## 1. Introduction

Nursing students’ readiness for clinical practicum is vital to nursing education. A clinical practicum allows students to apply their skills and professional judgment in real-world settings, explore potential career paths, and smoothly transition from academic learning to hands-on practice while guided by clinical instructors or preceptors. Student readiness is dependent on students’ familiarity with clinical settings, the strategic arrangement of theoretical and clinical courses within the nursing curriculum, whether in block, concurrent, or mixed-method formats [1], and students’ practical training during laboratory courses [2]. The use of simulation in the nursing laboratory is an effective tool to prepare students for their clinical practicum by providing them with opportunities to learn practical skills in a safe environment [2,3,4,5].

Students’ readiness can equip them with the essential skills to provide exemplary patient care standards. This preparedness facilitates the acquisition of clinical competence and critical thinking skills [6,7], provides hands-on experiences that bridge the gap between theoretical knowledge and its application in clinical settings [8], and prepares students for their future nursing careers in applying safe nursing care to real patients [9,10,11].

Several studies shed light on the experiences that undergraduate nursing students face during their transition to clinical practicum and hamper their readiness. These include a decrease in professional self-efficacy [12] due to inadequate practical skills and limited theoretical knowledge [13], a lack of self-confidence in working independently [14] the inability to recall knowledge, difficulty applying theory into practice, feelings of anxiety from students’ clinical practicum, lack of support from staff nurses, long working hours, and fear of making mistakes [15].

Nursing programs typically encompass theoretical and clinical courses [16]. In the United Arab Emirates (UAE), the Bachelor of Nursing program at the Nursing College is a three-and-a-half-year program, including three mandatory summer semesters. Each basic nursing course, the Clinical Health Assessment and Fundamentals of Nursing, has a co-requisite laboratory course to facilitate applying skills taught in the theory courses [17]. Moreover, nursing students must complete eight clinical courses starting in the second semester of their second year. However, these clinical courses lack co-requisite laboratory courses. Within this program, students receive clinical education from faculty members acting as clinical instructors and from preceptors or staff nurses employed in clinical settings such as hospitals or healthcare clinics.

As part of the UAE’s Emiratization strategy, the Abu Dhabi Health Services Company [18] collaborates with universities to promote nursing education among Emirati students [19]. This collaboration has led to a significant surge in Emirati students’ enrollment at the nursing college. However, the increase in students has not been met with a proportional rise in educators, resulting in a higher student-to-educator ratio in nursing laboratories, from 8 to 14 students per instructor. Consequently, instructors may struggle to allocate sufficient time to individual students to practice skills until mastery is achieved repeatedly, resulting in reduced skill development [20]. Feedback from clinical instructors and preceptors indicates that many student nurses exhibit deficiencies in both the knowledge and skills required for providing nursing care to actual patients, thereby raising questions about their readiness for performing in their clinical practicum [21]. Additionally, many students have expressed concerns regarding limited opportunities for individualized skill practice and heavy study loads, which hinder adequate preparation for clinical days.

Even though [22] explored the perceptions of senior students toward their readiness to perform different nursing skills upon graduation and found a lack of confidence in performing specific clinical procedures, research investigating the readiness of students for clinical practicum is scarce in the UAE. Therefore, addressing this knowledge gap in identifying the various factors that may influence nursing students’ readiness for practice is crucial as it holds implications for nursing curriculum development, clinical training protocols, faculty development initiatives, and policy-making efforts that aim to optimize the preparedness of future nursing professionals in the UAE.

This study aims to investigate undergraduate nursing students’ readiness for clinical practicum in the UAE by examining their perspectives, the variation in clinical study plans across different contexts, and the evidence gathered from clinical evaluations.

1. What explanations do the nursing students offer regarding their readiness for their clinical practicum?
2. How do variations in clinical nursing study plans across diverse contexts explain nursing students’ readiness for their clinical practicum?
3. What evidence of students’ readiness can be extracted from their clinical evaluations?

### Theoretical Framework

**Figure 1:**
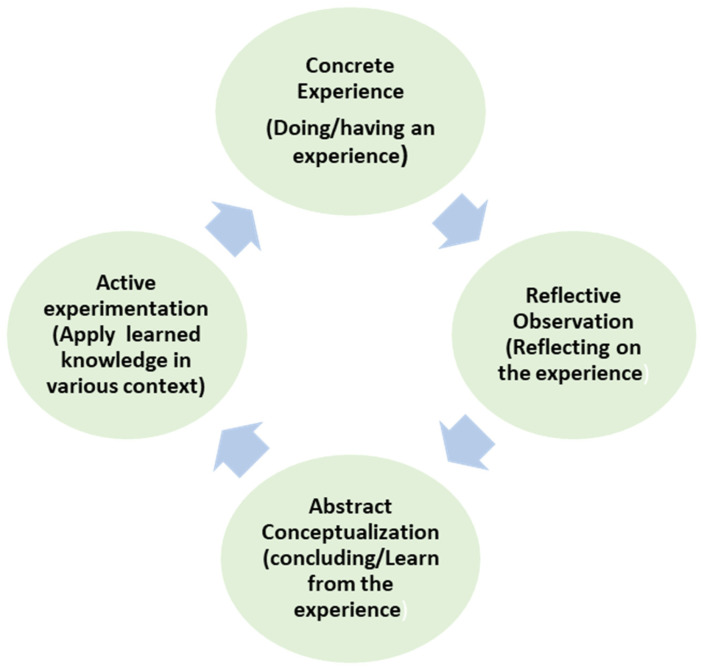
Kolb’s Experiential Learning Theory.

Kolb’s experiential learning theory serves as the theoretical framework for this study (Figure 1). Students’ readiness can be explained by Kolb’s experiential learning theory, which theorizes that learning occurs through four interrelated stages: concrete experience, reflective observation, abstract conceptualization, and active experimentation. These stages of learning can be integrated into the simulation scenarios that are used during laboratory courses to equip students with essential clinical skills for their practicum in clinical areas [23]. The effectiveness of this method is supported by studies on low-, medium-, and high-fidelity simulation scenarios [3,4,5,24]. Simulations effectively integrate all stages of experiential learning, starting with the concrete experience in which nursing students apply theoretical knowledge in a safe environment without causing any harm to patients [25]. This is followed by reflective observation, during which they review and make sense of their experiences to extract learning insights that could develop their critical thinking [26,27]. In the stage of abstract conceptualization, learners develop new ideas based on their reflections and refine their abstract understanding. Finally, active experimentation entails applying these new ideas in different contexts, such as hospital settings, and evaluating the outcomes to identify areas for improvement.

## 2. Materials and Methods

### 2.1. Research Approach

A qualitative approach was utilized to gain an in-depth understanding of students’ readiness for clinical practicum. In particular, the study employed a case study design. This design is useful to examine a “specific phenomenon or a problem in a particular setting” [28]. The authors followed the Consolidated Criteria for Reporting Qualitative Studies (COREQ). Table 1 presents a summary of the methodology.

### 2.2. Instruments

Semi-structured interviews were employed to collect data. The purpose of the semi-structured interviews was to answer the first research question, which is related to the participant’s description of their readiness for clinical practicum. The researchers developed an interview guide consisting of both open-ended and follow-up questions that served to obtain specific, organized, and complete information. The interview questions are:

- What is your opinion on how effective was your preparation for clinical practice by theoretical and lab courses?
  - What is your opinion about your readiness for clinical practice?
  - How do you prepare yourself for clinical practice?
  - What is your opinion on the effectiveness of lab sessions in preparing you for clinical practice, the lab session coverage of competencies that you need during the clinical placement, the opportunities you had in the lab to demonstrate procedures, and integrating lab sessions with theoretical nursing courses? Please rationalize your answer.

Moreover, document analysis was used, which is a systematic procedure to review or evaluate printed or electronic documents [29]. It encompasses examining and interpreting data to elicit meaning, gain understanding, develop empirical knowledge, and validate data obtained from semi-structured interviews [29], and was used to answer the second and third research questions. Two types of document analysis were used. First, the authors reviewed nursing study plans from top-ranked universities in the UAE, Lebanon, Jordan, the UK, Canada, Australia, and the USA. The official websites of these universities were accessed to obtain the original study plans to compare whether students receive adequate laboratory courses in preparation for their clinical practicum. This document analysis answered the second research question. Second, 217 clinical evaluation reports of third and fourth-year nursing students from the 4 campuses were also reviewed. This document analysis answered the third research question and was used to evaluate students’ knowledge and skills that could reflect their preparation from theoretical classes and laboratory sessions. Students’ overall clinical grades were also examined to assess their general clinical performance.

### 2.3. Study Population and Sampling Techniques

The study population consisted of undergraduate nursing students enrolled in the Bachelor of Nursing program across four campuses (Al Ain, Abu Dhabi, Ajman, and Al Dhafra). Most students were Emirati, while others came from various Arab countries, with their ages ranging between 19 and 23 years. Notably, all students in the nursing program were female at the time of the research due to cultural perceptions that discouraged Emirati males from pursuing nursing.

To select participants for the interview, the researchers employed purposeful sampling. Since most of the students were not expressive and felt too shy to talk to their instructors, we preferred to ask all third and fourth-year clinical instructors from the four campuses to recommend three to four of their most vocal students. They recommended a total of 17 students. We approached the selected students to participate in the study and sought their approval. Only 13 Emirati students agreed to participate.

Table 2 outlines the participants’ demographic data. Students were named using the ‘S’ letter, which is accompanied by a number; S2, S5, S8, S9, S11, S12, and S13 were third-year students, while S1, S3, S4, and S7 were fourth-year students.

### 2.4. Reliability and Validity of Semi-Structured Interviews

The researcher collaborated with three qualitative research experts to validate the interview questions. Some modifications were made based on their feedback [30]. To minimize bias, the researcher refrained from unnecessary speech or interruption during interviews [30]. An external auditor possessing a Ph.D and qualitative research expertise reviewed the transcription of data, and data analysis and interpretation, to ensure the inter-rater and inter-coder reliability as well as the trustworthiness of the data [30,31]. Transcription was also shared with the participants for accuracy confirmation, a technique known as member check [31].

Regarding the validity and potential biases in the clinical evaluation documents, one of the researchers with access to the archive approached the documents with complete objectivity, disregarding any identifying information such as students’ names or grades. The documents were stored in a locked cabinet and returned to the archive promptly after the researcher completed the review.

### 2.5. Data Collection and Procedure

Data collection began at the end of the clinical practicum to ensure that the participants, particularly third-year students, had adequately engaged with the clinical practicum. Students interested in participating in the study were asked to choose a convenient date and time for their interview. The participants were interviewed online via Zoom video calls by one of the researchers, who did not work at the college. The interviews were audio recorded with participant permission, each lasting around 30 minutes. Saturation was reached at 13 interviews, with the last few interviews yielding no new insights.

For the first document analysis, the researchers conducted an internet-based review of high-ranking bachelor’s nursing programs in the following regions: the UAE, Lebanon, Jordan, the UK, Canada, Australia, and the USA. However, program information was not consistently available on all university websites, which limited the inclusion of some institutions in the analysis. Eleven universities finally agreed to be involved: New York University, University of Alabama, Pennsylvania State University (USA), University of Toronto, McMaster University (Canada), University of Manchester (UK), Griffith University (Australia), American University of Beirut (Lebanon), Jordan University of Science and Technology (Jordan), Higher College of Technology, and University of Sharjah (UAE).

The second document analysis was undertaken by one of the researchers who worked at the UAE nursing college. The researcher obtained permission from the academic coordinator in each of the four campuses to access the student clinical evaluation reports, which were held in the archive room. A sample of 217 recent reports for year three and year four students was randomly selected. These reports were completed in the academic year 2021–2022.

### 2.6. Data Analysis

Thematic analysis was used to analyze the data obtained from the semi-structured interviews [31]. First, the recordings were transcribed verbatim and then saved in a Word document. The researchers repeatedly read the data to gain an overall understanding of the content and identify the main ideas. These ideas were coded, and these codes were organized into categories based on their similarities and differences. Related categories were then clustered under themes. Finally, the established themes were refined and organized to facilitate an in-depth description of these themes and interpretation of their meaning.

The first document analysis employed content analysis to examine a sample of 11 local, regional, and international nursing study plans. This analysis aimed to determine current trends in nursing education, focusing on whether the study plans included co-requisite or prerequisite laboratory courses.

The second document analysis also utilized content analysis of the clinical evaluation reports for 106 third-year and 109 fourth-year students. This analysis assessed their knowledge of clinical procedures, pharmacology, and pathophysiology, and their performance levels in clinical skills. These areas are essential for nursing care and medication administration. Pathophysiology, for instance, is crucial for understanding health concepts, disease processes, and responses to medication and care. Students’ performance was assessed on four levels: excellent, very good, satisfactory, and unsatisfactory. Excellent performance indicates meeting all objectives independently. Very good means meeting objectives with minimal guidance and timely task completion. Satisfactory means meeting most objectives with occasional guidance but requiring more time. Unsatisfactory includes failure to apply knowledge, unsafe skill execution, ignoring feedback, excessive absences, and unprofessional behavior [32]. The analysis also assessed the students’ clinical grades according to five grade range levels.

## 3. Results

### 3.1. Results of the Semi-Structured Interviews

**Figure 2:**
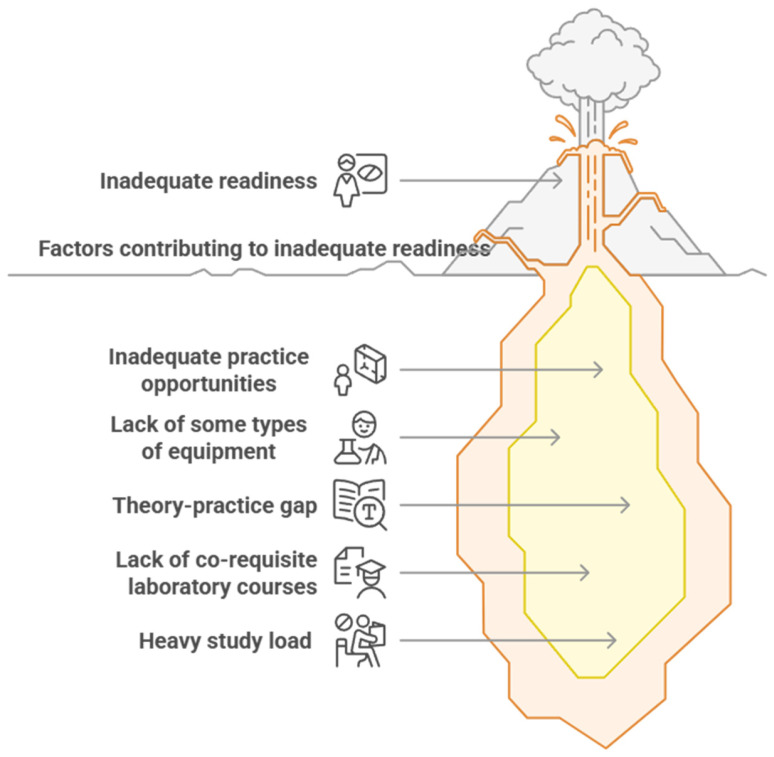
Findings of the thematic analysis.

The findings related to readiness for clinical practicum address research question number one. As shown in Figure 2, two main themes were extracted—incomplete readiness for clinical practicum and factors contributing to incomplete readiness—with five sub-themes emerging from the second theme.

#### 3.1.1. Incomplete Readiness for Clinical Practicum

All the participants were well-oriented to their clinical areas by their clinical instructors, preceptors, or nurse managers. However, 11 students (S1, S2, S3, S4, S5, S7, S8, S9, S11, S12, and S13) felt only partially ready for clinical practicum, with more than half of the third-year students and one-third of the fourth-year students reporting this. Some participants (S3, S4, S6, S7, S8, S9, and S12) noted losing nursing skills acquired in the first semester of the second academic year due to a gap before starting their clinical practicum in the second semester. This issue persisted in the third and fourth years and was attributed to the lack of co-requisite laboratory components in the clinical practicum courses.

#### 3.1.2. Factors Contributing to Incomplete Readiness

**Inadequate Practice Opportunities**

Some participants (S4, S6, and S10) noted having ample opportunities to practice procedures in the nursing laboratory. S10 explained that these sessions prepared her for clinical practicum, as repetitive practice under instructor guidance led to competency. However, most participants (S3, S5, S6, S8, S9, S11, S12, and S13) complained of inadequate practice opportunities in the laboratory courses. The inadequate opportunities were due to the short duration of lab sessions (S3, S8, S9, S11, S12, S13), large class sizes (S9), and ineffective online laboratory sessions during COVID-19 due to the absence of hands-on experience (S5, S12). S5 added that some instructors focused too much on explaining procedures rather than for hands-on practice, and S11 noted that the students face difficulty learning many nursing procedures during the clinical practicum due to the lack of cooperation from the staff nurses.

As a result, the participants (S2, S3, S5, S7, S8) felt the need for more practice opportunities in the lab, especially at the beginning of each academic year (S3). S8 emphasized, “Nursing is about practicing, practicing, and practicing. If I don’t practice, I will somehow lose my skills, even if I watch a million videos.” S9 agreed that having more demonstrations in the laboratory would prevent forgetting procedures during the clinical practicum. S2 suggested forming smaller laboratory groups of four to five students to enhance practice and discussion opportunities. S5 also proposed limiting theoretical explanations to lecture time and focusing laboratory sessions on practical demonstrations and re-demonstrations of procedures.

**Lack of some types of equipment**

Another issue was the lack of some types of equipment in the laboratory. S12 stated, “I couldn’t learn how to use the ECG machine or intravenous pumps because they weren’t available. The instructor only explained the procedures and showed pictures, but I never practiced on a manikin”, since high-fidelity manikins were defective (S1 and S7). In a similar manner, the lack of simulation experience deprived students of being prepared for critical care nursing skills (S10). S4 shared a similar opinion. S1 and S7 added that the outdated laboratory devices did not match modern hospital equipment, thus making the sessions ineffective.

**Theory and practice gap**

An additional issue raised was the gap between theory and practice. S6 found it challenging to care for patients with medical conditions, topics that might not yet be covered in the theoretical part of the Medical-Surgical Nursing course. This issue arose because the theory and clinical components of the course were taught simultaneously. As a result, S6 suggested that completing the theoretical part before starting the clinical practicum would be more beneficial, as this would allow her to apply the necessary knowledge during her clinical training and dedicate more time and effort to meet the requirements of the clinical practicum (S5). This suggestion for change was also raised by S3, S5, S11, and S12.

**Lack of co-requisite laboratory courses**

The absence of co-requisite laboratory courses was seen as a barrier to readiness for clinical practice. Participants stressed the need for a new co-requisite laboratory course for each clinical practicum course to allow students to practice procedures commonly encountered in clinical settings (S6), recall previous laboratory course content (S6 and S11), enhance their self-confidence when providing nursing care to patients (S7 and S8), and receive feedback on their performance before treating actual patients (S10).

Regarding the theory classes of the corresponding laboratory courses, the participants felt these courses provided adequate knowledge about nursing procedures. However, they emphasized the need for practical demonstrations in both laboratory and clinical settings to solidify skills and improve their self-confidence (S5 and S6). They noted that theory courses did not enhance decision-making and critical thinking skills, which are crucial for clinical practicum (S7). Moreover, these courses often lacked clinical scenarios (S5 and S7), which are necessary to connect theory with practice (S6).

**Heavy study load or inadequate preparation for clinical day**

Some participants (S4, S5, and S6) prepared for clinical days by reading materials, watching videos (S5 and S6), and researching information about drugs (S4). S7 used to write her daily objectives to obtain feedback from the instructor. S3 only read about patients’ conditions for case presentations. However, most participants (S1, S2, S3, S7, S8, S9, S10, S11, and S12) did not regularly prepare at home and only occasionally reviewed medications or procedures (S2 and S11). S8 noted that reviewing procedures was ineffective as staff nurses performed them differently than what they learned. Observing and asking nurses questions about the procedure was better for learning (S8 and S9). Moreover, S1, S2, S7, and S9 attributed the lack of preparation to a high study load, a major stressor for S11. S12 explained, “The theory courses were very demanding because they required me to study the assigned materials, complete assignments on time, and prepare myself for quizzes and examinations.” S2 and S1 suggested reducing theory content to allow more time for clinical preparation.

### 3.2. Results of Document Analysis

#### 3.2.1. First Document Analysis

The first document analysis addressed the second research question by identifying current trends in nursing education. This included focusing on study plans and determining whether they incorporate co-requisite or prerequisite laboratory courses.

Table 3 shows that only four selected American and Australian universities have co-requisite laboratory courses for all clinical practicum courses. The seven remaining universities or colleges cover clinical competencies in the laboratory component of basic nursing courses offered one or two semesters earlier. All major nursing courses (Medical-Surgical, Pediatric, Maternity, Mental Health, Community Health, and Critical Care Nursing) have co-requisite clinical practicum courses.

The structure of all documents’ study plans shows that the theory component of basic nursing courses (Clinical Health Assessment and Fundamentals of Nursing) has a co-requisite laboratory course. However, four universities (University of Toronto, University of Manchester, Higher College of Technology, and Griffith University) have one or two basic nursing courses with a co-requisite clinical practicum course. Only the Higher College of Technology offers a co-requisite practicum course for Fundamentals of Nursing. These universities start practicum courses in the first year, while others, including New York University and the University of Pennsylvania, start in the second or third year. At the UAE nursing college, second-year students begin practicum courses concurrent with a medical-surgical nursing course in the second semester.

#### 3.2.2. Second Document Analysis

The second document analysis aimed to address the third research question. This analysis presented evidence of students’ readiness extracted from their clinical evaluations.

As shown in Table 4, the review results for year three clinical evaluation samples showed that 77.9%, 57.9%, 75.5%, and 46.76% of students displayed very good or excellent knowledge of clinical procedure, pathophysiology, pharmacology, and clinical skills, respectively.

As shown in Table 5, the review results for the year four clinical evaluation samples showed that 89.4%, 60.4%, 80.1%, and 70.7% of students displayed very good or excellent knowledge of clinical procedures, pathophysiology, pharmacology, and clinical skills, respectively. The overall clinical grade of each of the three students from the selected sample was reviewed.

Table 6 shows that 8.8% of the grades were equal to or greater than 90%. A total of 44.4% fell in the range between 80% and 89%, while 31.10% of the grades ranged between 70% and 79%. Only 11.1% of the grades were between 60% and 69%. Grades below 60% were not found.

Concerning year four students, Table 7 shows that 32% of the grades were equal to or greater than 90%; 36% of the grades fell between 80% and 89%, while 20% of the grades ranged between 70% and 79%. Only 1.3% of the grades were between 60% and 69%. No grades below 60% were found.

## 4. Discussion

### 4.1. Discussion of the Semi-Structured Interviews

The results show that participants were well-oriented in their clinical placement. However, they complained of partial readiness for clinical practicum [33,34], and this could negatively impact students’ professional efficacy and confidence in working independently [13,35]. As such, improving their self-efficacy through supportive bonds with mentors, peer encouragement, and independent skills practice is important to achieve desired proficiency levels [14]. 

One key factor attributed to the limited practice opportunities is the scheduling of introductory laboratory courses in the first semester of the second year of the nursing program. Clinical courses are scheduled for the second semester of the second year, the third year, and the first semester of the fourth year, and these lack co-requisite laboratory components. Consequently, students face difficulty in recalling and applying knowledge learned earlier in their studies [36]. Thus, introducing new co-requisite laboratory courses alongside clinical courses could better prepare students by providing opportunities for them to consolidate their clinical skills and comprehend their rationale before applying them in real settings, thereby helping them to develop competence and confidence as practitioners [2,37].

Moreover, the inadequate laboratory practice with too short sessions, a high student-to-instructor ratio (1:14), and online delivery sessions during the COVID-19 pandemic, affected students’ skills development due to limited or a lack of hands-on experiences [38]. During the COVID-19 pandemic, online platforms were used to maintain the continuity of students’ learning. However, there was a gap in their practical skills learning, which affected their readiness for clinical practicum. Several reasons contributed to creating this gap. Not all students were committed to attending the online sessions without being distracted during the unsupervised time. Students had restricted access to hands-on activities, and clinical instructors had less chance to closely evaluate students’ performance, where some mistakes might go unnoticed due to difficulty assessing non-verbal cues. The unreliable internet access hindered the participation of students and instructors, and many instructors lacked sufficient training and support to use these tools effectively. The virtual simulation is seen as inadequate for teaching hands-on skills and professional behaviors [39].

Laboratory courses are crucial for developing students’ knowledge, skills, and attitudes [2]. Therefore, maintaining an appropriate ratio of instructors to students, 1 to 8 or 1 to 10 [2], and increasing session frequency to improve competency and allow more time for practice and discussions, are suggested. Moreover, adopting a combination of face-to-face training, with strict standard precautions and online simulation, is also recommended to enhance nursing skills [38].

The inadequate hands-on experience was also attributed to defective manikins that prevented students from using medium- and high-fidelity simulations. Such simulations are effective for the preparation of students for their clinical practicum [3,5] by enhancing nursing skills, self-confidence, and critical thinking, as well as narrowing the theory-practice gap [3,40,41]. When students use low-quality or faulty manikins during simulations, they often face difficulties that are attributed to the simulation’s lack of human features, such as facial expressions and body language. Students may feel nervous or unsure when using unfamiliar or unpredictable manikins, which hinders their ability to practice important communication and relationship-building skills. Students may not take the activity seriously, making it harder to stay focused and learn effectively. This can lower their confidence and make them less willing to fully take part in the simulation [42].

**Figure 3:**
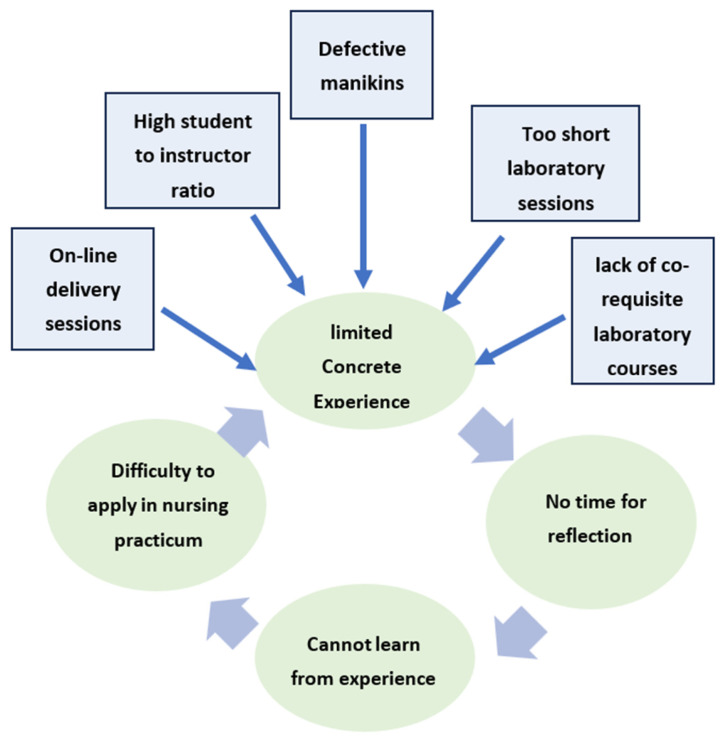
Limited concrete experiences alter the first stage of Kolb’s cycle.

Kolb’s experiential learning theory (1984) supports using simulation sessions by emphasizing reflective sessions for improvement. However, the limited concrete experience, as shown in Figure 3, from inadequate laboratory practices left students with no time for reflective observation to extract learning insights. This lack of reflection deprives students of identifying their mistakes and developing new ideas to refine their abstract understanding, and this would, in turn, confine their ability to apply knowledge in real-world clinical practice, such as hospital settings. This highlights the need for providing necessary laboratory resources and repairing high-fidelity manikins to prepare students for nursing practicum [5].

**Figure 4:**
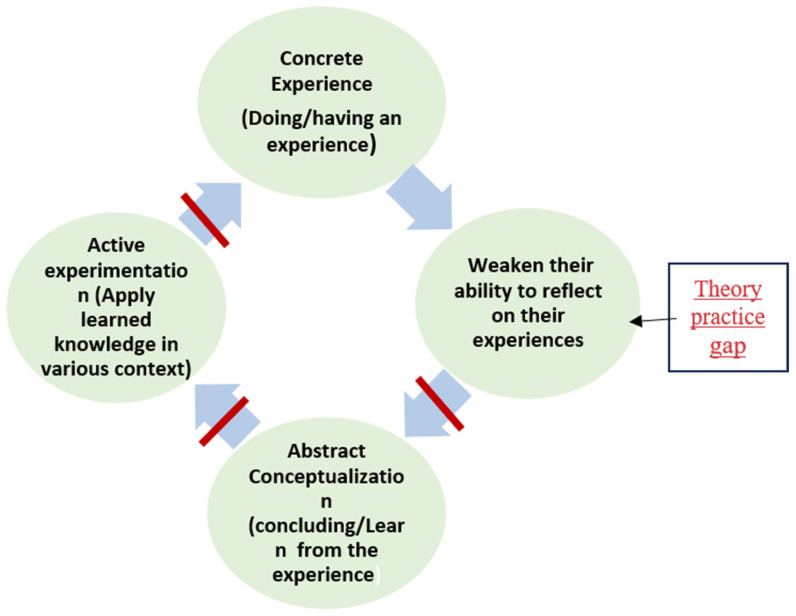
The theory-practice gap alters the second stage of Kolb’s cycle.

The lack of clinical scenarios in theory courses [35] limits students’ opportunities to apply practical knowledge into practice, which hinders the development of key competencies such as decision-making, critical thinking, problem-solving, and self-directed learning [43,44]. It could also weaken their ability to reflect on experiences encountered during clinical rotations. Incorporating clinical scenarios through case studies has been shown to foster reflective thinking on complex educational issues [45]. Without this support, students may find it difficult to fulfill the second stage of Kolb’s experiential learning theory, which emphasizes reflection and meaning making from experience to generate learning insights [26,27]. Thereby, this gap interrupts the flow of experiential learning, as shown in Figure 4.

The concurrent pattern of theory and clinical courses left some students unprepared for clinical practicum, as theoretical concepts were not yet covered [46]. Some participating students preferred the block pattern for better “assimilation”, “consolidation”, and “socialization” [47], in addition to enhancing a realistic sense of work and promoting a singular focus during clinical practicum [48]. The block pattern could suit senior students with self-confidence and good clinical skills. However, the concurrent pattern could be more suitable for first-year students as it helps integrate theory with practice, promotes reflection, and helps students adapt to clinical environments [1,48]. Therefore, a combined approach is recommended [1].

**Figure 5:**
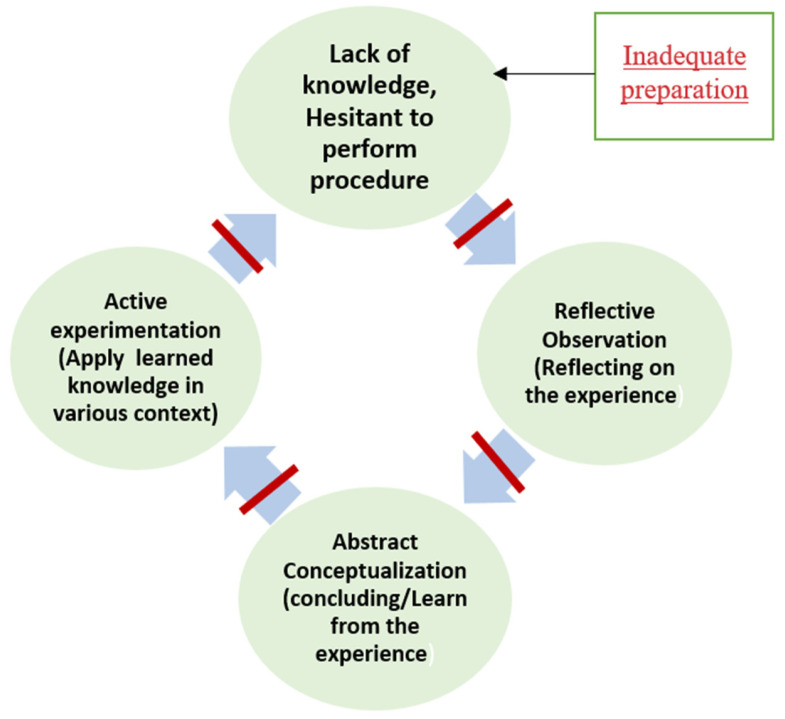
Inadequate preparation alters the first stage of Kolb’s cycle.

Lastly, most students did not regularly prepare for clinical days due to their high study load, leading to a lack of knowledge about some nursing procedures. This lack of knowledge can make students hesitant to perform procedures out of fear of making mistakes [49]. Insufficient preparation could impact students’ skill development and cause stress related to a lack of self-confidence in applying nursing care [50]. Thereby, the first stage of Kolb’s experiential learning theory, concrete experience, cannot be initiated, which hinders the continuation of the experiential learning cycle, as shown in Figure 5. Some students proposed reducing theory course content to allocate more time for clinical preparation.

### 4.2. Discussion of Document Analysis

The analysis found that most universities and colleges follow a study plan similar to that of nursing colleges in terms of lacking co-requisite laboratory courses for clinical practicum courses. In the UAE nursing college, all laboratory courses occur in the second year. In the last two years, clinical courses have included intensive laboratory sessions only during the first two clinical days of each semester. Some students may need repeated practice, which is often not feasible in the limited time available, making it hard to memorize and competently perform nursing procedures. This raises concerns about the effectiveness of the current study plan structure and requires a re-evaluation of its impact on students’ readiness for clinical practicum. Safe and effective patient care requires sound judgment, deliberate planning, critical thinking, and decision-making skills, and not just performing skills [2]. Therefore, adopting the American and Australian approach of pairing practicum courses with laboratory courses may better prepare students for clinical practicum.

The review of clinical evaluation reports for third-year students mainly revealed very good or excellent knowledge of clinical procedures and pharmacology, likely due to their preparation efforts. However, some students showed unsatisfactory knowledge of clinical procedures and pathophysiology, and unsatisfactory performance in clinical skills. This indicates difficulty in applying knowledge to practice, potentially affecting the ability to provide quality care and suggesting a lack of readiness for the clinical practicum. This might be due to students limited clinical experience, as they only started practicum in the second semester of the second year. Moreover, the overall clinical grades suggest that most students perform satisfactorily to a good level, implying a generally good standard of competence. However, when referring to [32], the researchers noticed that some aspects of the clinical evaluation are measurable and can be objectively evaluated. Other aspects include items related to evaluating students’ attitudes and professional behaviors, which are more subjective and can possibly lead to bias and inflating clinical grades; thereby, potentially misrepresenting actual knowledge and skill levels. In such a situation, students might pass the course or even graduate from the program without fulfilling the necessary skills, which would lead to providing poor patient care. Ultimately, the credibility of the program and the graduating students will be negatively affected. Reference [51] more on this idea by indicating that grade inflation negatively impacts students, institutions, and society at large. For students, this can create a false sense of competence and decrease motivation to work harder, leaving them unprepared for future academic or professional challenges. For educational institutions, it can diminish their ability to accurately assess and differentiate student performance, leading to complications in admissions and hiring decisions. At a societal level, grade inflation can worsen inequality by giving unfair advantages to already privileged students in securing jobs.

A further revision of the clinical evaluation document is needed to ensure it accurately reflects students’ competencies. This may involve changing the criteria to emphasize measurable competencies and skills over subjective evaluations of attitudes and behaviors.

Most year four students demonstrated very good or excellent knowledge of clinical procedures and pharmacology, similar to year three students, but with a higher percentage achieving excellence. This improvement is likely to be due to increased exposure to theory and clinical courses. Despite better pharmacology knowledge, the unsatisfactory knowledge level of pathophysiology and performance of clinical skills among senior students is concerning and indicates that some are still not fully ready for clinical practicum. The overall clinical grades showed a high standard of competence, which was even better than in year three. Again, these grades might not accurately reflect their clinical skills due to including non-clinical factors in grading [31].

### 4.3. Strengths

The strength of this study goes back to addressing a crucial aspect of nursing education: students’ readiness for clinical practicum in the context of increasing student-to-educator ratios and limited hands-on practice opportunities. Such a topic can extend beyond the UAE as it offers valuable insights into common challenges faced by nursing education worldwide.

A distinctive strength of this research is document analysis, which compares UAE institutions’ nursing curricula with high-ranking universities at the local, regional, and global levels. An extensive search of the existing literature found no previous research that utilized this comparative approach. This unique methodology provides a comprehensive understanding of how curriculum structures impact students’ preparedness for clinical practicum, thereby filling a critical gap in the literature.

### 4.4. Limitations

The study is limited to investigating the undergraduate nursing students’ readiness for clinical practicum in only a nursing college with four branches. This sample may not represent the entire nursing student population in the UAE. Moreover, there is a possibility that some participants may have provided untruthful responses when answering the interview questions.

## 5. Conclusions

The study showed that many students felt only partially prepared, and this impacted their skill development and confidence. The laboratory was held in the first semester of year two, while all the clinical courses from the second semester in years two to four lacked co-requisite laboratory practice, leading to skills loss. Large class sizes and defective equipment further limited practice opportunities and switching to an online laboratory during COVID-19 reduced hands-on experiences. The heavy study load hindered adequate preparation, and the concurrent pattern of theory and clinical courses also posed challenges because theoretical concepts were not covered early enough.

Document analysis showed that most universities lacked co-requisite laboratory courses for practicum courses, unlike American and Australian institutions. Students at the UAE nursing college preferred adding co-requisite laboratory sessions, which could improve nursing students’ readiness for clinical practicum.

Clinical evaluation reports indicated that some year three and four students were not ready for clinical practicum due to poor clinical skills and pathophysiology knowledge. Year four students performed worse in clinical skills despite having more experience. These findings align with the interview results, highlighting deficiencies in the understanding of clinical procedures and pharmacology, and the performance of clinical skills.

### Recommendations for Further Research

Based on this study’s findings, future research could include a broader sample from all UAE nursing colleges for more robust findings and an examination of students’ perceptions of block versus concurrent theory and clinical courses. Additionally, exploring preceptors’ views on students’ readiness for clinical practicum would be beneficial.

## Figures and Tables

**Table 1:** A Summary of the study’s methodology.

Research Questions	Approach	Instrument	Data Analysis	Participants
1. What explanations do the nursing students offer regarding their readiness for their nursing practice?	Qualitative	Semi-structured interviews	Thematic analysis	A purposeful sample of 13 students
2. How do variations in clinical nursing study plans across diverse contexts impact nursing students’ readiness for their nursing practicum?		Document analysis	Content analysis	A sample of 11 national, regional, and international nursing study plans
3. What evidence of students’ readiness can be extracted from their clinical evaluations?		Document analysis	Document analysis	A sample of 217 students’ clinicalevaluations

**Table 2:** Nursing students’ demographic data.

Participants	Age	Year of Study	Campus	Nationality
S1	22	Fouth	Al Ain	Emirati
S2	19	Third	Ajman	Emirati
S3	23	Fourth	Abu Dhabi	Emirati
S4	22	Fourth	Al Dhafra	Emirati
S5	20	Third	Ajman	Jordanian
S6	21	Third	Al Ain	Emirati
S7	22	Fourth	Al Ain	Emirati
S8	20	Third	Al Dhafra	Emirati
S9	21	Third	Abu Dhabi	Emirati
S10	21	Third	Al Ain	Emirati
S11	21	Third	Abu Dhabi	Emirati
S12	21	Third	Ajman	Emirati
S13	20	Third	Abu Dhabi	Syrian

**Table 3:** Comparison of the structure of the study plans of selected universities/colleges.

University/College	Country	Co-requisite Lab Courses for Practicum Courses	Co-Requisite Practicum Courses for Major Nursing Courses	Co-Requisite Practicum Courses for Basic Nursing Courses	Start of Clinical Practicum
New York University	USA	Yes	Yes	No	Year 2
Pennsylvania University	USA	Yes	Yes	No	Year 3
University of Alabama	USA	Yes	Yes	No	Year 2
Griffith University	Australia	Yes	Yes	Yes	Year 1
University of Toronto	Canada	No	Yes	Yes	Year 1
University of Manchester	UK	No	Yes	Yes	Year 1
Higher College of Technology	UAE	No	Yes	Yes (Fundamentals of Nursing only)	Year 1
Macmaster University	Canada	No	Yes	No	Year 2
Jordan University	Jordan	No	Yes	No	Year 2
Sharjah University	UAE	No	Yes	No	Year 3
American University of Beirut	Lebanon	No	Yes	No	Year 3
Nursing College	UAE	No	Yes	No	Year 2/ second semester

**Table 4:** Percentages of year three students according to their performance level.

	Unsatisfactory Performance	Satisfactory Performance	Excellent or Very Good Performance
Clinical procedure knowledge	17.7%	4.4%	77.9%
Pathophysiology knowledge	35.5%	6.6%	57.9%
Pharmacology knowledge	11.1%	13.33%	75.57%
Clinical skills	8.8%	44.44%	46.76%

**Table 5:** Percentages of year four students according to their performance level.

	Unsatisfactory Performance	Satisfactory Performance	Excellent or Very Good Performance
Clinical procedure knowledge	6.6%	4.0%	89.4%
Pathophysiology knowledge	25.3%	5.3%	60.4%
Pharmacology knowledge	10.6%	9.3%	80.1%
Clinical skills	16%	13.3%	70.7%

**Table 6:** The percentages of clinical grades of year three students according to five grade level ranges.

Grade Range	Percentage of the Grade
Equals or greater than 90%	8.8%
80–89%	44.4%
70–79%	31.1%
60–69 %	11.10%
Less than 60%	0%

**Table 7:** The percentages of clinical grades for year four students according to five grade level ranges.

Grade Range	Percentage of the Grade
Equals or greater than 90%	32%
80–89%	36%
70–79%	20%
60–69 %	1.30%
Less than 60%	0%

## Data Availability

Most of the data analyzed are presented in the Results section of the article. Additional data can be provided upon request from the corresponding author.

## Public Involvement Statement

There was no public involvement in any aspect of this research.

## Guidelines and Standards Statement

This manuscript was drafted against the Consolidated Criteria for Reporting Qualitative Research (COREQ), a 32-item checklist for interviews and focus groups. https://www.equator-network.org/ accessed on 12 April 2024.
